# TIMP-1 inhibits proliferation and osteogenic differentiation of hBMSCs through Wnt/β-catenin signaling

**DOI:** 10.1042/BSR20181290

**Published:** 2019-01-08

**Authors:** Tangzhao Liang, Wenling Gao, Lei Zhu, Jianhua Ren, Hui Yao, Kun Wang, Dehai Shi

**Affiliations:** 1 Department of Orthopaedic Surgery, the Third Affiliated Hospital of Sun Yat-sen University, No. 600, Tianhe Road, 510630 Guangzhou, China; 2 Department of Orthodontics, Hospital of Stomatology, Sun Yat-sen University, No. 56, Lingyuan West Road, 510060 Guangzhou, China; 3 Department of Plastic and Reconstructive Surgery, the Third Affiliated Hospital of Sun Yat-sen University, No. 600, Tianhe Road, 510630 Guangzhou, China

**Keywords:** Cell proliferation, Mesenchymal stem cells, Osteogenic differentiation, Tissue inhibitor of metalloproteinase-1, Wnt/β-catenin signaling

## Abstract

The present study aimed to evaluate the effect of tissue inhibitor of metalloproteinase-1 (TIMP-1) on the proliferation and osteogenic differentiation potential of human bone marrow-derived MSCs (hBMSCs). hBMSCs with stable TIMP-1 overexpression or TIMP-1 knockdown were generated. Osteogenic differentiation was assessed by Alizarin Red S staining, alkaline phosphatase (ALP) activity and expression of specific markers. Compared with the vehicle controls, TIMP-1 knockdown significantly promoted the growth of hBMSCs. TIMP-1 knockdown up-regulated β-catenin and cyclin D1 proteins. During osteogenic differentiation, TIMP-1 knockdown elevated the deposition of calcium nodules, ALP activity and the mRNA levels of the osteogenic markers sex determining region Y-box 9 (Sox9), CCAAT-enhancer-binding protein and peroxisome proliferator-activated receptor γ. During osteogenic differentiation, TIMP-1 knockdown significantly enhanced the up-regulation of osteocalcin proteins. Meanwhile, TIMP-1 overexpression attenuated the Wnt/activator Wnt3a-induced up-regulation cyclin D1 and Runt-related transcription factor 2 (RUNX-2) (during osteogenic differentiation) proteins, while TIMP-1 knockdown restored the inhibitor Dickkopf 1-induced inhibition effect on the expression of β-catenin, cyclin D1 and RUNX-2. TIMP-1 plays a negative regulatory role in the proliferation and osteogenesis of hBMSCs, at least partially, through Wnt/β-catenin signaling.

## Introduction

Bone marrow-derived mesenchymal stem cells (BMSCs) are commonly adopted in various cell-based therapy for tissue repair and regeneration [1]. It has been demonstrated that BMSCs transplantation enhances bone regeneration in both pre-clinical [2] and clinical study [3]. Previous study has shown that MSCs can move to the fracture sites and differentiate into osteocytes for bone healing [4]. In this respect, understanding the mechanism of osteogenic differentiation of MSCs is helpful for developing the strategy of hMSCs therapy for bone defects or fractures.

During the early stage of osteogenic differentiation of MSCs, accumulated extracellular matrix (ECM) forms 3D nodules at the sites of mineral deposition [5]. Matrix metalloproteinases (MMPs) are a family of proteolytic enzymes involved in remodeling of ECM, and their enzyme activity is regulated by binding with tissue inhibitors of matrix metalloproteinases (TIMPs) [6]. Hence, TIMPs play an important role in the balance of ECM synthesis and degradation. Tissue inhibitor of metalloproteinase-1 (TIMP-1) is a multifunctional protein and ubiquitously expresses in a variety of human cells and tissues and participates in a variety of biological processes [7].

In addition to regulating MMPs, TIMP-1 has been shown to participate in the regulation of various MMP-independent biological activities, including cell growth and survival [8], cell migration [9], differentiation [10] and apoptosis [11]. Accumulating evidence has suggested that TIMP-1 possesses the potent growth-promoting activity for several types of cells [12–14]. Nevertheless, the growth-inhibitory effect of TIMP-1 has also been reported in epithelial cells [15] and rheumatoid synovial fibroblasts [16]. In addition, Egea et al. [17] have demonstrated that knockdown of TIMP-1 enhances proliferation of MSCs, which also suggests a negative effect of TIMP-1 on proliferation. These findings indicate that for different cell types, TIMP-1 may have different regulations on the cell proliferation.

It has been demonstrated that TIMP-1 plays an important role in the osteogenesis of osteoblasts and MSCs [18–20]. Grässel et al. [21] report that during osteochondrogenic differentiation of rat adult mesenchymal progenitor cells, TIMP-1 is highly expressed. Likewise, Molloy et al. [22] have demonstrated that MSCs secrete a high level of TIMP-1 during differentiation into osteoblasts. These observations indicate that TIMP-1 is a positive regulator of osteogenic differentiation in osteoblasts or MSCs. On the contrary, Schiltz et al. [23] report that TIMP-1 overexpression reduces and delays osteoblastic differentiation in mice osteoblasts. Egea et al. [17] show that knockdown of TIMP-1 promotes osteogenic differentiation of MSCs, suggesting TIMP-1 has a negative regulation effect on the osteogenic differentiation. Therefore, the regulatory effect of TIMP-1 on the osteogenesis remains controversial.

To further elucidate the effect of TIMP-1 on MSCs, the present study aimed to investigate the regulatory effect of TIMP-1 on the proliferation and osteogenic differentiation of human BMSCs (hBMSCs).

## Materials and methods

### Isolation, cultivation and characterization of hBMSCs

Bone marrow sample was collected from the drill holes of the pedicle during the operation of spine internal fixation of ten patients with traumatic lumbar spondylolisthesis at our center from March 2014 to June 2014. The present study was approved by the Institutional Review Board (IRB) of the First Affiliated Hospital of Sun Yat-sen University, and all the procedures were performed in accordance with the IRB guidance. Written informed consent was obtained from each patient.

hBMSCs were isolated and cultured as described in our previous studies [24,25]. The surface markers of hBMSCs were characterized using flow cytometry analysis. For induction of osteogenic differentiation, hBMSCs were cultured in the differentiation medium (DMEM containing 10% FCS, 100 nm dexamethasone 10 nm b-glycerophosphate and 50 mg/ml ascorbate-2-phosphate) [26].The chondrogenic and adipogenic differentiation potentials of hBMSCs were assessed as previously described [24,25].

### Construction of TIMP-1 expressing lentivirus vector and transduction

To generate a pDest_puro_ vector, the puromycin resistance encoding sequence was used to replace both the blasticidin resistance encoding sequence and the bacterial EM7 promoter of lentiviral 2k7bsd vector. In order to generate entry vectors, human TIMP-1 cDNA (648 bp) flanked by attB sites was PCR-amplified from BAC clone GM090230 (GENECHEM Biotech Company, Shanghai, China) and cloned into Pdonr^™^ 221 (Invitrogen, U.S.A.) by utilizing the Gateway BP recombination reaction according to the manufacturer’s protocol. The EF1a promoter and heGFP cDNA were cloned into Pdonr^™^ P4-P1R (Invitrogen) and Pdonr^™^ P2r-P3 (Invitrogen) by the same method. The resulting vectors, pDown-TIMP-1, pUp-EF1a and pTail-IRES-heGFP, were then recombined into the Dest_puro_ vector according to the protocol for LR recombination reaction using the Gateway LR plus clonase enzyme mix (Invitrogen). The constructed lentiviral vector was designated as pLV/Final-puro-EF1a-TIMP-1-hrGFP. In the present study, we used the pDest_puro_ vector with heGFP gene inserted as the vehicle control.

The lentiviral particles were prepared by transient cotransfection of 293FT cells with the lentiviral vectors and ViraPower^™^ Lentiviral packaging mix (Invitrogen) using Lipofectamine 2000 (Invitrogen). Three days after transfection, viral particles were harvested from the medium, filtered through 0.45 µm pore-sized polyethersulfone membrane and concentrated by ultracentrifugation at 50000×***g***, 4°C for 120 min. For lentiviral transduction, hMSCs were washed with PBS and dissociated into single cells by 0.25% trypsin and were replated with lentiviral particles and 8 mg/ml polybrene (Sigma, U.S.A.). The medium was changed to fresh culture medium after infection for 12 h. After twice transduction, puromycin (Sigma) was added to the culture medium at a concentration of 1 to 5 μg/ml and maintained for 5 days.

### Construction of shRNA -TIMP-1 vector and transfection

The shRNA target sequences were as follows: TIMP-1-shRNA-F: 5-GATCCCCAAGATGTATAAAGGGTTCCAA TTCAAGAGATTGGAACCCTTTATACATCTTTTTTTGGAAA; TIMP-1-shRNA-R: AGCTTTTCCAAAAAAAGATGTATAAAGGGTTCCAATCTCTTGAATTGGAACCCTTTATACATCTTGGG. Plasmid pGenesil-1 consists of the eGFP cDNA (GFP as an indicator of transfection efficiency), prokaryotic selection marker Kanamycin and eukaryotic selection marker Neomycin. The shRNA-TIMP-1 sequence was constructed into the pGenesil-1 vectors. The shRNA-TIMP-1 plasmid was transiently transfected into MSCs using Lipofectamine 2000 reagent. Briefly, 2 × 10^4^ MSCs were seeded in a well of 24-well plate (500 µl) and was transfected with 50 ng plasmid DNA [27]. After transfection, the cells were incubated at 37°C for 24 h. Forty-eight hours after transfection, cells were observed under a fluorescence microscope and selected with 500 μg/ml final concentration of G418. Selection medium was changed every 3 days for 3 weeks.

### Proliferation assay

hBMSCs proliferation was determined by 2-(2-methoxy-4-nitrophenyl)-3-(4-nitrophenyl)-5-(2,4-disulfo-phenyl)-2H-tetrazolium, monosodium salt (WST-8) assay (Cell Counting Kit-8, CCK-8, Dojindo, Japan) according to the manufacturer’s protocol. The absorbance at 450 nm was measured using a microplate reader (Thermo Scientific, U.S.A.).

### Western blot

Western blot was performed as previously described [28]. Specific antibodies including monoclonal TIMP-1, osteocalcin (OC), antibodies (Santa Cruz), Runt-related transcription factor 2 (RUNX-2), cyclin D1 and β-catenin (Cell Signaling Technology, U.S.A.) were all used in the Western blot analysis. The final concentration of all primary antibodies was 1 µg/ml [28].

### Real-time RT-PCR

Total RNA was extracted from hBMSCs with Trizol reagent (Invitrogen, U.S.A.). cDNA synthesis was performed with 1 μg of total RNA with the RevertAid™ Premium First Strand cDNA Synthesis Kit (Fermentas, U.S.A.) according to the manufacturer’s protocol. The expressions of RUNX-2, type I collagen (COL-1), CCAAT-enhancer-binding protein (C/EBP), peroxisome proliferator-activated receptor γ (PPARγ) and sex determining region Y-box 9 (SOX-9) were detected with real-time PCR kit (TAKARA, Japan) with specific primers, using ABI Prism 7700 SDS apparatus (Applied Biosystems, U.S.A.). The primer sequences were as follows: SOX9-F: GGAGATGAAATCTGTTCTGGGAATG; SOX9-R: TGAAGGTTAACTGCTGGTGTTCTGA; COL1A1-F: CCCGGGTTTCAGAGACAACTTC; COL1A1-R: TCCACATGCTTTATTCCAGCAATC; RUNX2-F: CACTGGCGCTGCAACAAGA; RUNX2-R: CATTCCGGAGCTCAGCAGAATAA; PPARG-F: TGGAATTAGATGACAGCGACTTGG; PPARG-R: CTGGAGCAGCTTGGCAAACA; C/EBPα-F: TGGACAAGAACAGCAACGAG; C/EBPα-R: TTGTCACTGGTCAGCTCCAG; GAPDH-F: GCACCGTCAAGGCTGAGAAC; GAPDH-R: TGGTGAAGACGCCAGTGGA. The mRNA fold changes were calculated on quadruple experiments by using the comparative critical threshold cycle (*C*_T_) 2^–ΔΔ*C*^_T_ value. Six plates replicated were used for each group, and all reactions were performed in triplicate.

### Statistical analysis

All values are presented as the mean ± standard deviation. Data were analyzed using the Student’s *t*-test or a general linear model two-way ANOVA with Tukey post hoc test to compare between groups. A *P*-Value < 0.05 was considered statistically significant.

## Results

### Isolation and culture human BMSCs

**Figure 1:**
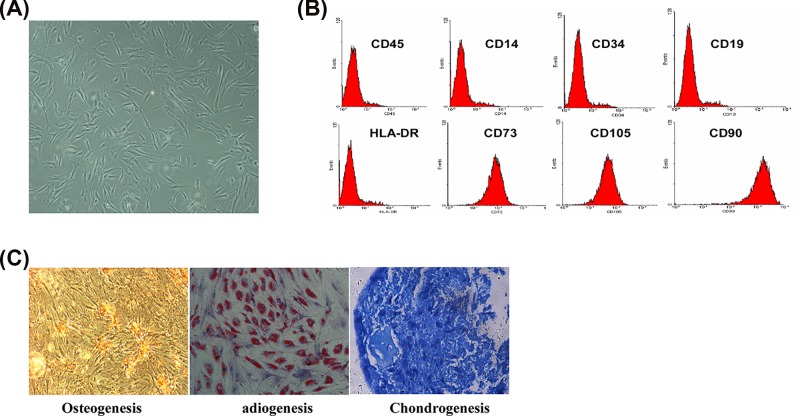
Characterization of the surface markers and multilineage differentiation potentials of hBMSCs (**A**) On Day 7 after isolation, the adherent hBMSCs (passage 3) exhibited the long-spindle fibroblastic morphology. (**B**) Characterization of hBMSCs cell surface markers by flow cytometry, including CD14, CD19, CD34, CD45, CD73, CD105, CD90 and HLA-DR. (**C**) Evaluation of multilineage differentiation potentials of hBMSCs. Adipogenesis was assessed by Oil Red O staining. Osteogenesis was determined by Alizarin Red S staining. Chondrogenesis was determined by Toluidine Blue staining.

hBMSCs were isolated and cultured from human bone marrow sample (Figure 1A). Flow cytometry analysis showed that hBMSCs were positive for the surface markers CD105, CD73 and CD90 but negative for the hematopoietic (CD45, CD14, CD19 and CD34) markers and human leukocyte antigen (HLA-DR) (Figure 1B). In addition, the osteogenic, adipogenic and chondrogenic potentials of hBMSCs were demonstrated by Alizarin Red S, Oil Red O, and Toluidine Blue staining (Figure 1C), respectively. These data suggested that the cultured hBMSCs possessed the characteristics of MSCs.

### The effect of TIMP-1 on hBMSC proliferation and Wnt/β-catenin signaling

**Figure 2:**
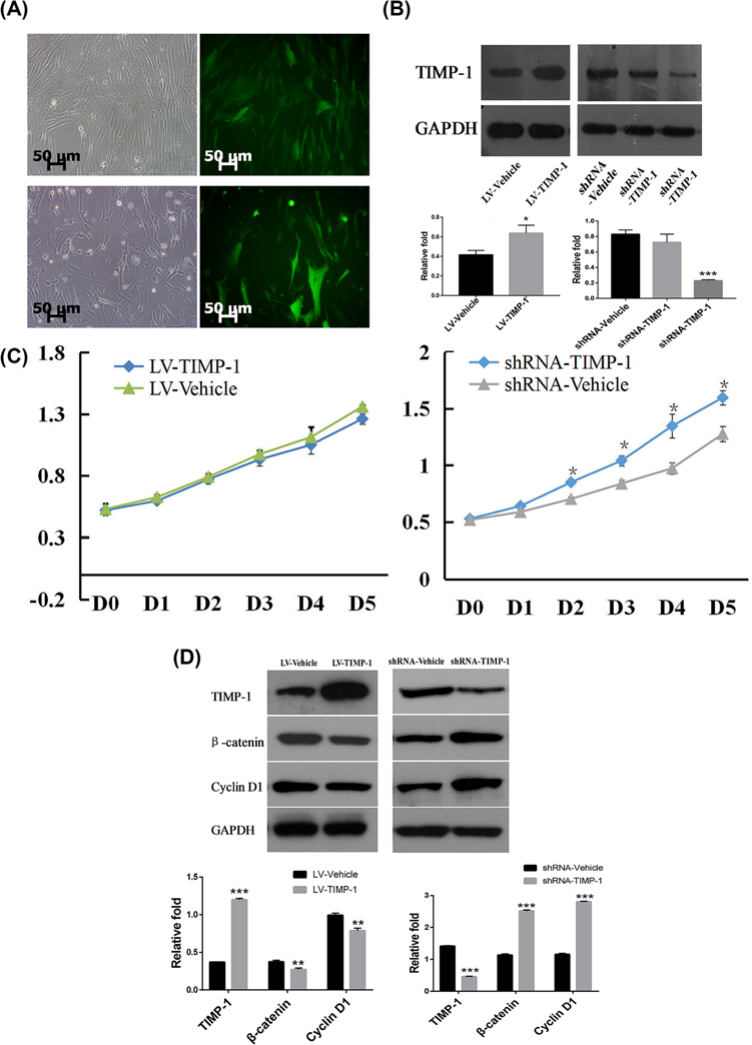
The effect of TIMP-1 on hBMSC proliferation and Wnt/β-catenin signaling (**A**) Microscopic morphology of TIMP-1 overexpression and TIMP-1 knockdown hBMSCs. After 48 h, the transduced/transfected cells were observed using a fluorescence microscopy. The green fluorescence indicated that GFP protein expressed by the coding sequence inserted in the recombinant vectors. (**B**) TIMP-1 protein levels in TIMP-1 overexpression and TIMP-1 knockdown hBMSCs were determined by Western blot at 72 h after the start of the experiment. (**C**) The proliferation of hBMSCs TIMP-1 overexpression and TIMP-1 knockdown hBMSCs was determined by WST-8 assay for 5 days (**D**) The protein levels of TIMP-1, β-catenin and Cyclin D1 in TIMP-1 overexpression and TIMP-1 knockdown hBMSCs were determined by Western blot at 72 h after the start of the proliferation experiment. *P<0.05, **P<0.01, ***P<0.001, compared with the corresponding vehicle control.

To evaluate the effect of TIMP-1 on the physiological functions of hBMSC, TIMP-1 stable overexpressing (LV-TIMP-1) hBMSCs and TIMP-1 stable knockdown (shRNA- TIMP-1) hBMSCs were generated. The majority of cells displayed green fluorescence, suggesting that the target gene was transfected and expressed successfully (Figure 2A). Western blot showed that TIMP-1 protein levels significantly increased in LV-TIMP-1 hBMSCs and decreased in shRNA-TIMP-1 hBMSCs (both *P* < 0.05, Figure 2B).

The effect of TIMP-1 on the growth of hBMSCs was investigated. The results demonstrated that the proliferation of LV-TIMP-1 hBMSCs slightly decreased from day 3 (D3) to D5, while the proliferation of shRNA-TIMP-1 hBMSCs significantly increased from D3 to D5 as compared with the vehicle control (all *P*<0.05, Figure 2C).

Next, we addressed the effect of TIMP-1 on Wnt/β-catenin signaling. Both the proteins of β-catenin and its target gene Cyclin D1 were significantly down-regulated in LV-TIMP-1 hBMSCs but significantly up-regulated in shRNA-TIMP-1 hBMSCs (all *P*<0.01, Figure 2D), suggesting that TIMP-1 had a negative effect on the proliferation and Wnt/β-catenin signaling of hBMSCs.

### Effects of TIMP-1 gene on hBMSC multilineage differentiation

**Figure 3:**
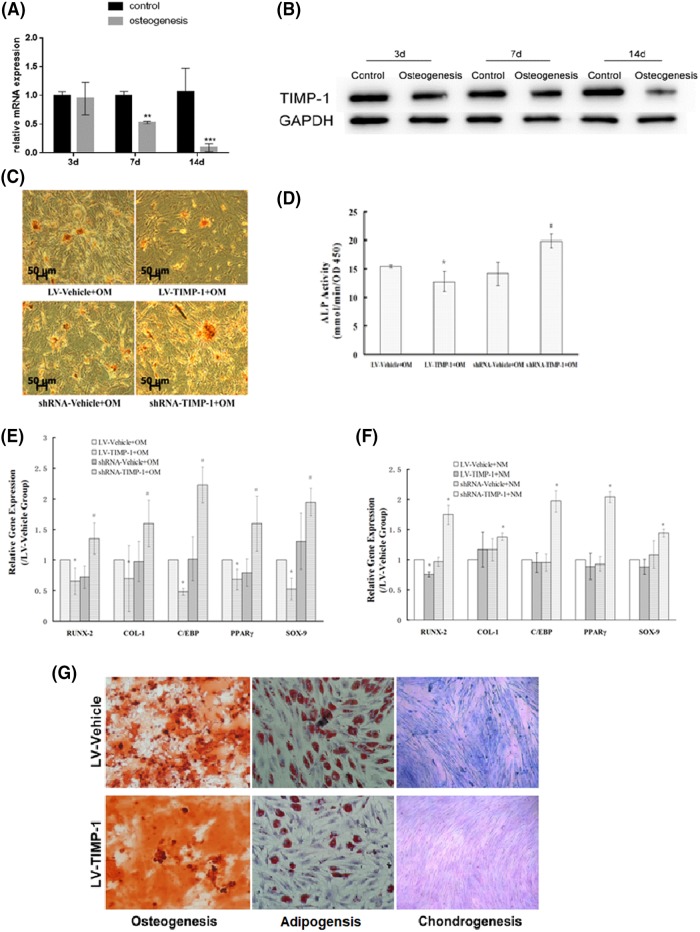
The effects of TIMP-1 on hBMSC osteogenic differentiation The RNA (**A**) and protein (**B**) levels of TIMP-1 were determined at 3, 7 and 14 days after the osteogenic differentiation was determined by RT-PCR or Western blot, respectively. Data were presented as means ± SEM in the bar chart; *N*=6 for each group; **P<0.05, ***P<0.01. (**C**) After osteogenic induction for 14 days, all induction groups manifested a varying degree of calcium deposits positively stained with Alizarin Red S for osteogenetic differentiation. (**D**) ALP activity is evaluated after cultured in osteogenic induction medium (OM). (**E**) after osteogenetic induction (OM) for 14 days, the mRNA expression of RUNX-2 (an osteogenesis-related transcription factor) and osteoblastic differentiation-related marker collagen type I (COL-1a), SOX-9 (a chondrogenesis-related gene) and C/EBP and PPARγ (both adipogenesis-related genes) were determined by qRT-PCR. (**F**) In normal culture medium (NM), the expression of these differentiation markers was assessed. (**G**) The osteogenic, adipogenic and chondrogenic differentiation were compared between the LV-TIMP-1 hBMSCs and LV-Vehicle control. *P<0.05, compared with the corresponding vehicle control.

The effect of TIMP-1 on hBMSC osteogenic differentiation was assessed. RT-PCR and Western blot were performed to determine the RNA and protein levels of TIMP-1 at 3, 7 and 14 days after the osteogenic differentiation, respectively. The results showed that the TIMP-1 RNA level was significantly down-regulated at 7 days (*P*<0.01) and 14 days (*P*<0.001) after osteogenic differentiation (Figure 3A). The change in the TIMP-1 protein during osteogenic differentiation showed a similar decreasing trend (Figure 3B). After osteogenic induction for 14 days, LV-TIMP-1 hBMSCs had decreased deposition of calcium nodules (Figure 3C) and a significantly reduced ALP activity (Figure 3D, *P*<0.05) as compared with LV-Vehicle control. By contrast, shRNA-TIMP-1 hBMSCs had an increased deposition of calcium nodules (Figure 3C) and significantly elevated ALP activity as compared with vehicle control (Figure 3D, *P*<0.05). In addition, qRT-PCR demonstrated that TIMP-1 overexpression significantly down-regulated the mRNA levels of RUNX-2 (an osteogenesis-related transcription factor) and osteoblastic differentiation-related marker collagen type I (COL-1a), while TIMP-1 knockdown significantly down-regulated these two genes as compared with their vehicle controls (all *P*<0.05).

Meanwhile, after osteogenic induction for 14 days, SOX-9 (a chondrogenesis-related gene) and C/EBP and PPARγ (both adipogenesis-related genes) were all significantly down-regulated in LV-TIMP-1 hBMSCs, while these three genes were significantly up-regulated in shRNA-TIMP-1 hBMSCs as compared with their vehicle controls (all *P*<0.05, Figure 3E).

In the normal medium, TIMP-1 knockdown significantly up-regulated the level of all five genes compared with the control groups (all *P*<0.05), whereas TIMP-1 overexpression had little or no effect (Figure 3F). Moreover, morphological evidence showed that compared with the vehicle control, LV-TIMP-1 hBMSCs exhibited relatively poor multilineage differentiation, including osteogenic, adipogenic and chondrogenic differentiation (Figure 3G), which was consistent with the findings of RT-PCR. These data indicated that TIMP-1 had a negative effect on the multilineage differentiation of hBMSCs.

### Wnt/β-catenin signaling involved in the inhibitory effects of TIMP-1 on hBMSC osteogenic differentiation

**Figure 4:**
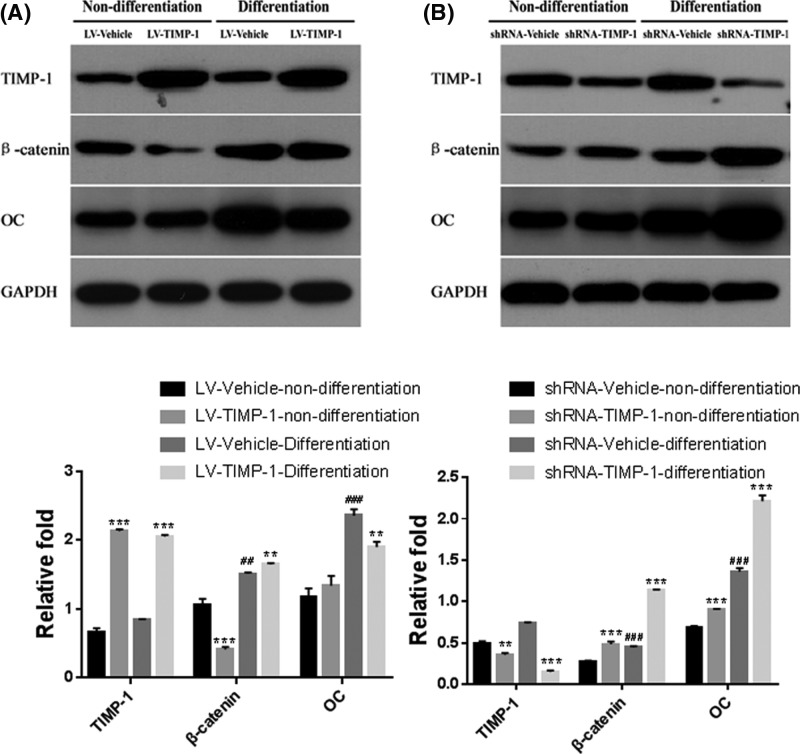
Wnt/β-catenin signaling involved in the inhibitory effects of TIMP-1 on hBMSC osteogenic differentiation The endogenous protein levels of TIMP-1, β-catenin and OC in TIMP-1 overexpression hBMSCs (**A**) and TIMP-1 knockdown hBMSCs (**B**) under normal culture medium (non-differentiation) or osteogenic induction medium (cultured for 14 days) were determined by Western blot. **P<0.01, ***P<0.001, compared with the corresponding vehicle control. ^##^*P*<0.01, ^###^*P*<0.001, compared with the corresponding non-differentiation group.

Since TIMP-1 had a negative effect on Wnt/β-catenin signaling, we examined if Wnt/β-catenin signaling participated in the TIMP-1 regulation on ECM’s metabolism during hBMSC osteogenic differentiation. After osteogenic differentiation for 14 days, the protein levels of β-catenin and osteocalcin (OC) were significantly elevated (all *P*<0.01, Figure 4A,B). However, TIMP-1 overexpression significantly attenuated the up-regulation of OC during osteogenic differentiation (Figure 4A), while TIMP-1 knockdown significantly enhanced the up-regulation of β-catenin and OC levels (both *P*<0.001, Figure 4B). These findings indicated that the Wnt/β-catenin signaling was involved in the inhibitory effects of TIMP-1 on hBMSC osteogenic differentiation.

**Figure 5:**
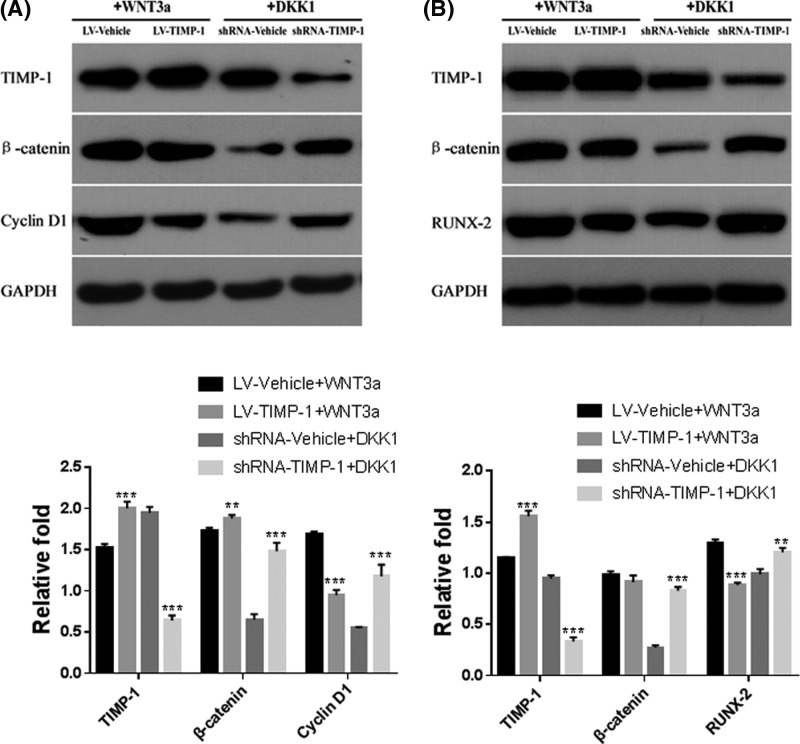
TIMP-1 inhibited the proliferation and osteogenic differentiation potential of hBMSC through Wnt/β-catenin signaling (**A**) In normal culture medium, the protein levels of TIMP-1, β-catenin and cyclin D1 were determined in TIMP-1 overexpression hBMSCs (treated with 150 ng/ml Wnt/β-catenin signaling activator Wnt3a for 3 days) and TIMP-1 knockdown hBMSCs (treated with 100 ng/ml Wnt/β-catenin signaling inhibitor Dickkopf 1 [DKK1] for 3 days). (**B**) After osteogenic induction for 7 days, the protein levels of TIMP-1, β-catenin and RUNX-2 were determined in TIMP-1 overexpression hBMSCs (treated with 150 ng/ml Wnt/β-catenin signaling activator Wnt3a for 7 days) and TIMP-1 knockdown hBMSCs (treated with 100 ng/ml Wnt/β-catenin signaling inhibitor Dickkopf 1 [DKK1] for 7 days). **P<0.01, ***P<0.001, compared with the corresponding vehicle control.

To further investigate the relationship between Wnt/β-catenin signaling and TIMP-1 expression on hBMSC proliferation and osteogenesis, the activator (Wnt3a) and the inhibitor (Dickkopf 1 [DKK1]) of Wnt/β-catenin signaling were used. As shown in Figure 5A, TIMP-1 overexpression significantly attenuated the Wnt3a-induced up-regulation of cyclin D1 expression (*P*<0.001, Figure 5A). By contrast, TIMP-1 knockdown significantly restored the DKK1-induced inhibition effect on β-catenin and cyclin D1 expression (both *P*<0.001, Figure 5A).

At 7 days after osteogenic differentiation, TIMP-1 overexpression suppressed the Wnt3a-induced up-regulation of RUNX-2 proteins (*P*<0.001 Figure 5B), while TIMP-1 knockdown attenuated the DKK-1-induced inhibition effect on β-catenin and RUNX-2 expression (both *P*<0.01, Figure 5B). Taken together, these findings revealed that TIMP-1 plays an important role in the proliferation and osteogenesis of hBMSC through Wnt/β-catenin signaling.

### TCF4 knockdown down-regulated mRNA expressions of TIMP-1 and RUNX-2

**Figure 6:**
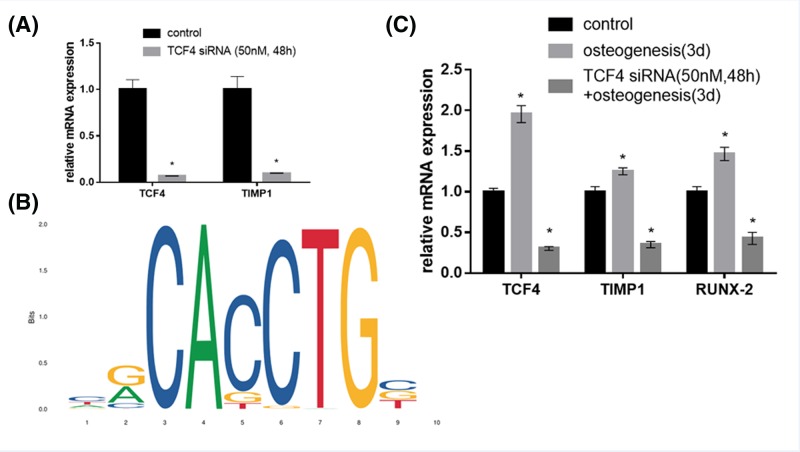
Knockdown of TCF4 by siRNA down-regulated mRNA levels of TIMP-1 and RUNX3 (**A**) mRNA expressions of TCF4 and TIMP-1 in TCF4-knockdown hBMSCs. (**B**) Sequence logo of TCF4 contained an E-Box DNA element. (**C**) mRNA expressions of TCF4, TIMP-1 and RUNX3 in TCF4-knockdown hBMSCs. *P<0.05, compared with the control group.

It has been shown that TCF4 play a crucial role in the Wnt/β-catenin signaling and cellular development [29,30]. We investigated the effect of TCF4 knockdown on the TIMP-1 expression and osteogenic differentiation in hBMSCs. As shown in Figure 6A, TCF4 knockdown (siRNA sequence: GGTCTAGAAATGGAGGACA) down-regulated TIMP1 mRNA in hBMSCs as compared with the negative control.

Previous study has shown that TCF4 is a conserved bHLH transcription factor that binds E-box sequences in the promoters and enhancers of certain genes [31]. We, therefore, analyzed the transcription-factor binding site predictions between TIMP-1 and TCF4 by using JASPAR database (http://jaspar.genereg.net) [32]. As shown in Figure 6B, the sequence logo of TCF4 contained an E-Box DNA element that was recognized as an important TIMP-1-binding site [31]. Two putative TCF4-binding sites were predicted on the TIMP-1 promoter (Table 1).

**Table 1:** Transcription-factor binding site of TCF4 predicted on TIMP-1 promoter region

Matrix ID	Name	Score	Relative score	Sequence ID	Start	End	Strand	Predicted sequence
MA0830.1	TCF4	5.17495	0.866575	NG_012533.1:5001-9501	1075	1084	+	ATCAGCTGGG
MA0830.1	TCF4	4.75575	0.860164	NG_012533.1:5001-9501	84	93	+	CGCACCCGCT

TCF4 knockdown significantly down-regulated mRNA expression of osteogenic gene RUNX-2 at 72 h after osteogenic induction (Figure 6C), indicating osteogenesis differentiation of hBMSCs was inhibited.

## Discussion

In the present study, we investigated the effect of TIMP-1 on the proliferation and osteogenic differentiation potential of hBMSCs by using lentiviral-mediated overexpression and shRNA interference technique. The results showed that TIMP-1 knockdown significantly increased the growth of hBMSCs as compared with the vehicle control. TIMP-1 overexpression significantly down-regulated the protein levels of β-catenin and cyclin D1, but TIMP-1 knockdown up-regulated these two proteins. In addition, during osteogenic differentiation, TIMP-1 overexpression reduced the deposition of calcium nodules and ALP activity, and the mRNA levels of SOX-9, C/EBP and PPARγ, whereas TIMP-1 knockdown exhibited the opposite effects. During osteogenic differentiation, TIMP-1 overexpression significantly attenuated the up-regulation of OC, whereas TIMP-1 knockdown displayed the opposite trends. Meanwhile, TIMP-1 overexpression attenuated the Wnt3a-induced up-regulation of cyclin D1 and RUNX-2, while TIMP-1 knockdown restored the DKK1-induced inhibition on the expression of β-catenin, cyclin D1 and RUNX-2 (during osteogenic differentiation). Taken together, these results suggested that TIMP-1 has a negative regulatory role in the proliferation and osteogenesis of hBMSC through Wnt/β-catenin signaling.

The growth-stimulating effect of TIMP-1 has been described in a variety of cell types [12–14]. Contrary to these reports, Taube et al. [28] have reported that TIMP-1 reduces the growth of human breast epithelial cells by down-regulation of cyclin D1 and up-regulation of p27, resulting in G1 arrest. Our results also showed a growth-inhibitory effect of TIMP-1 on hBMSCs, which is consistent with a previous report [17]. These observations reflect the complexities of TIMP-1 effects [7]. Regarding differentiation of hBMSCs, TIMP-1 has been shown to be a positive regulator of osteogenic differentiation in osteoblasts and MSCs [21,22]. However, our study revealed that overexpression of TIMP-1 knockdown enhanced hBMSC osteogenic capability. Our findings are consistent with Egea et al.’s study, which also demonstrated that knockdown of TIMP-1 promotes osteogenic differentiation of MSCs [17]. Our study provides the data from TIMP-1 overexpression hBMSCs, which further strengthens the evidence supporting the negative regulatory effect of TIMP-1 on the proliferation and osteogenic differentiation of hBMSCs. Notably, our data showed that TIMP-1 overexpression in hBMSCs down-regulated the expression of adipogenesis-related genes C/EBP and PPARγ and chondrogenesis-related gene SOX-9 when cultured in the normal medium. When cultured in the osteogenic induction medium, TIMP-1 overexpression still inhibited the expression levels of C/EBP and SOX-9. These findings implied that TIMP-1 may suppress the expression of multilineage differentiation markers to maintain the stemness of hBMSCs. Further study is required to explore the biological meaning of the inhibitory effect of TIMP-1 on these differentiation markers.

It has been reported that the Wnt/β-catenin signaling plays a positive role in the proliferation and osteogenic differentiation of MSCs [33,34]. Our results showed that TIMP-1 overexpression attenuated the Wnt3a (a Wnt/β-catenin activator)-induced up-regulation of cyclin D1 expression, whereas TIMP-1 knockdown restored the DKK1 (a Wnt/β-catenin inhibitor)-induced inhibition effect on β-catenin and cyclin D1 expression. After osteogenic differentiation for 7 days, TIMP-1 overexpression suppressed the Wnt3a-induced up-regulation of RUNX-2 proteins, while TIMP-1 knockdown attenuated the DKK-1-induced inhibition effect on β-catenin and RUNX-2 expression. Taken together, these findings indicated that the inhibition effects of TIMP-1 on cell proliferation and osteogenic differentiation are dependent on, at least partially, Wnt/β-catenin signaling. Consistently, Egea et al. [17] also revealed that knockdown of TIMP-1 in MSCs with siRNA up-regulated the stability, nuclear translocation and promoter activity of β-catenin, demonstrating that TIMP-1 is an inhibitor of Wnt/β-catenin signaling pathway. Egea et al.’s study has demonstrated that TIMP-1 exerts its negative regulatory effect on the growth and osteogenic differentiation of hMSCs through binding to the CD63 surface receptor, resulting in down-regulation of let-7f miRNA and promoting the degradation of β-catenin [17]. In addition to its important role in the TIMP-1-mediated interference of the Wnt/β-catenin signal pathway, CD63 receptor also regulates the activity of the phosphatidylinositol 3-kinase/Akt [35], focal adhesion kinase [36] and Src [37] signal pathways. Whether these CD63 downstream signalings are involved in the inhibition effect of TIMP-1 on the proliferation and osteogenic differentiation of hBMSCs is worth further investigating.

It is known that activation of the Wnt/β-catenin pathway can suppress the degradation of cytosolic β-catenin by inducing saturation on the APC/Axin1 destruction complex [38,39]. Subsequently, the accumulated β-catenin translocates to the nucleus and associated with LEF-1/TCF transcription factors, which is a key step in activating the transcription of Wnt target genes [29,30]. On the other hand, Yoshioka et al. [40] have illustrated that mouse TIMP-1 proximal promoter can be repressed by a dominant-negative TCF4 in the immature undifferentiated mesenchymal cells. Considering the important role of TCF4 in the Wnt/β-catenin signaling and cellular development, we investigated the effect of TCF4 knockdown on the TIMP-1 expression and osteogenic differentiation in hBMSCs. Our results showed that TCF4 knockdown down-regulated mRNA expressions of TIMP-1 and RUNX-2 at 72 h after osteogenic induction (Figure 6C), indicating osteogenesis differentiation of hBMSCs was inhibited. It has been suggested that gene promoter region containing a TCF4-binding site may directly interact with the β-catenin/TCF complex upon Wnt signal activation [41]. Our observations suggested that TCF4 may directly bind to the TIMP-1 promoter and regulate osteogenesis differentiation of hBMSCs. However, the detailed molecular mechanism remains to be further investigated.

There are still some limitations of the present study. First, the *in vitro* findings should be validated in an *in vivo* model. In addition, the biological meaning of the inhibitory effect of TIMP-1 on differentiation markers requires further study. Moreover, the detailed mechanism underlying Wnt/β-catenin signaling involved in TIMP-1 effect on the proliferation and osteogenic differentiation remains to be further investigated. All these limitations should be addressed in the following study.

Overall, our findings demonstrate that TIMP-1 is a negative regulator of the growth and osteogenic differentiation of hBMSCs, at least partially, through inhibiting Wnt/β-catenin signaling. Our findings should be helpful for better understanding the mechanism of growth and osteogenic differentiation of hBMSCs.

## Abbreviations

ALP: alkaline phosphatase
BMSC: bone marrow-derived mesenchymal stem cell
ECM: extracellular matrix
hBMSC: human bone marrow-derived MSC
HLA-DR: human leukocyte antigen
MMP: matrix metalloproteinase
OC: osteocalcin
RUNX-2: Runt-related transcription factor 2
TIMP-1: tissue inhibitor of metalloproteinase-1
